# Description of the putative mature larva of the Neotropical genus *Stenhelmoides* Grouvelle (Coleoptera: Elmidae)

**DOI:** 10.1038/s41598-020-62978-w

**Published:** 2020-04-10

**Authors:** Marcela González-Córdoba, Nicolás Rafael Martínez-Román, María del Carmen Zúñiga, Verónica Manzo, Miguel Archangelsky

**Affiliations:** 1 Grupo de Investigaciones Entomológicas GIE, Universidad del Valle, Departamento de Biología, Calle 13 No. 100-00, Cali, 72000 Valle del Cauca Colombia; 2 Laboratorio de Investigaciones en Ecología y Sistemática Animal LIESA, Centro de Investigación Esquel de Montaña y Estepa Patagónica CIEMEP (CONICET - UNPSJB), Roca 780, Esquel, 9200 Chubut Argentina; 3 Instituto de Biodiversidad Neotropical IBN (UNT - CONICET), Crisóstomo Álvarez 722, San Miguel de Tucumán, 4000 Tucumán Argentina

**Keywords:** Biodiversity, Entomology, Freshwater ecology, Limnology

## Abstract

*Stenhelmoides* Grouvelle is a Neotropical genus of Elmidae and although it exhibits a wide distribution, until now no larvae had been associated with adults of this genus. Larvae, very likely belonging to this genus, were associated based on co-occurrence with adults. Eleven larvae and nine adults were examined from ten localities at altitudes between 30 and 682 m a.s.l. in the Andean, Caribbean and Pacific regions in Colombia. Mature larvae of the *Stenhelmoides rufulus* (Hinton) are described and illustrated for the first time. A larval diagnosis for the genus is presented; body shape, distribution and form of setae and number of pleural sclerites are diagnostic characters for larvae of this genus. Comparative notes with similar larvae of other Elmidae genera are presented. The existing generic keys are updated to include larvae of *Stenhelmoides*. Comments on distribution and habitat of *Stenhelmoides* larvae are also provided. This work contributes to enhance the knowledge of Neotropical larvae, which have been described for only approximately 56% of genera and 8% of species of Elmidae.

## Introduction

Riffle beetles (Coleoptera, Elmidae) are common dwellers of running waters and constitute an important component of benthic macroinvertebrate communities^1^. They are considered effective bioindicators of water quality and commonly used in ecological studies. Both larvae and adults are found in freshwater ecosystems; however, the immature stages of Elmidae are poorly known. Even though elmid larval knowledge is not complete, several improvements have been achieved in recent years. In the first place, several keys that allow identification of Elmidae larvae have been produced^2–5^. Description of larvae belonging to several genera and species have also been published: *Austrelmis catamarcensis* Manzo & Archangelsky, 2015^6^, *Austrelmis talampayensis* Manzo & Archangelsky 2015^6^, *Austrelmis uaik* Martínez-Román, Archangelsky & Manzo, 2017^7^*, Austrolimnius nyctelioides* (Germain, 1892)^8^ and A. *elatus* Hinton, 1941^8^*, Luchoelmis kapenkemkensis* Archangelsky & Brand, 2014^9^*, Macrelmis pubescens* (Grouvelle, 1889)^10^*, Neblinagena mira* Čiampor, Čiamporová-Zaťovičová & Kodada, 2017^11^*, Phanoceroides aquaticus* Hinton, 1939^12^, *P. fernandesi* Laššová, 2016^12^, *Stethelmis shepardi* Martínez-Román, Manzo & Archangelsky, 2019^13^, *Typhloelmis caroline* Barr, 2015^14^, *T. finegan* Barr, 2015^14^ and *T. sanfelipe* Barr, 2015^14^. Finally, molecular techniques have been useful to perform larval and adult associations^11,15,16^, these were first used for assigning the larva of *Hedyselmis opis* Hinton, 1976^17^

A better larval knowledge and a higher taxonomic resolution can support the use of riffle beetles in a wider range of fields such as biogeography, and significantly enhance their use as environmental bioindicators in ecological studies^18^. Furthermore, larval characters in phylogenetic studies can successfully be used to solve relationships among taxa^19,20^.

The Neotropical region is characterized by a high-level of endemism and great diversity of Elmidae with around 50 genera and 500 species^21^. One of those Neotropical genera is *Stenhelmoides* Grouvelle, 1908, with 15 described species and a wide geographical distribution. It is recorded in 15 countries from southern Mexico to Brazil, at altitudes between 100 and 2900 m a.s.l.^21,22^. However, the wide distribution of this genus is primarily a reflection of occurrence of a single species; *Stenhelmoides rufulus* (Hinton, 1934). The remaining 14 species are present in South America, mainly in lowlands of the Amazon region^22,23^.

Adult *Stenhelmoides* are distinguished from other Elmidae by the following combination of characters: dorsal tomentum over pronotum and elytra, with an oval area without tomentum in the middle of pronotum, the lack of carinae or depressions on pronotum and the presence of granulose rows on elytra without forming carinae^22^.

Prior to this contribution, the larva of *Stenhelmoides* was undescribed. The same can be said for several other genera of Neotropical riffle beetles, therefore a detailed revision of distributional data was performed in order to rule out the possibility that this larva could belong to another genus. In Colombia 29 genera of Elmidae have been reported (Table 1), but only ten of those genera have undescribed larvae. All of them, except for *Notelmis*, *Onychelmis* and *Stenhelmoides* are found east of the Andes (Table 1). Larvae of *Notelmis* and *Onychelmis* have been associated with adults by molecular techniques (unpublished data), additionally these larvae and their adults are small and unlike *Stenhelmoides*, they are frequent and abundant in sampled streams, something that facilitated their association. On the other hand, *Stenhelmoides* is uncommon and the studied material comes from a few museum specimens, and no fresh material has been available to perform molecular associations, or even to try to rear them. We also considered all elmid genera found in the same locations where *Stenhelmoides* specimens were collected, and all of them had known larvae. Taking in account the co-occurrence of these larvae with adults of *Stenhelmoides rufulus*, the larvae here described can be assigned to this species with a good degree of confidence.

**Table 1:** Elmidae genera present in the Neotropics (from Mexico to Argentina), Colombia to west and east of the Andes and bordering countries.

Genus		larva	Colombia		Bordering countries				
			West	East	Brazil	Ecuador	Panama	Peru	Venezuela
*Amazonopsis*								X	X
*Anommatelmis*									
*Austrelmis*		+	X			X		X	
*Austrolimnius*		+	X	X	X	X	X	X	X
*Cylloepus*		+	X	X	X	X	X	X	X
*Disersus*		+	X	X		X	X	X	X
*Elachistelmis*									
*Epodelmis*				X					
*Gyrelmis*		*		X	X				X
*Heterelmis*		+	X	X	X	X	X	X	X
*Hexacylloepus*		+	X	X	X	X	X	X	X
*Hexanchorus*		+	X	X	X	X	X	X	X
*Hintonelmis*				X	X	X		X	X
*Hispaniolara*		+							
*Holcelmis*				X					
*Huleechius*		+	X	X	X				
*Hydora*		+							
*Hypsilara*									X
*Ictelmis*						X			
*Jolyelmis*		+							X
*Lemalelmis*									
*Luchoelmis*		+							
*Macrelmis*		+	X	X	X	X	X	X	X
*Microcylloepus*		+	X	X	X		X	X	X
*Neblinagena*		+							X
*Neocylloepus*		+	X	X			X		X
*Neoelmis*		+	X	X	X	X	X	X	X
*Neolimnius*				X	X				X
*Notelmis*		*	X	X		X	X	X	
*Onychelmis*		*	X	X		X	X	X	
*Oolimnius*					X				
*Pagelmis*					X	X			
*Phanoceroides*		+		X	X				X
*Phanocerus*		+	X	X	X	X	X	X	X
*Pharceonus*		+	X	X	X	X	X	X	X
*Pilielmis*				X	X				X
*Portelmis*				X	X	X		X	
*Potamophilops*		+			X				X
*Pseudodisersus*		+	X	X		X	X		
*Roraima*		+							X
*Stegoelmis*		+		X	X	X		X	X
*Stenhelmoides*		+	X	X	X	X	X	X	X
*Stethelmis*		+							
*Tolmerelmis*					X				
*Tolriolus*		+							
*Tyletelmis*				X	X				X
*Xenelmis*		+	X	X	X	X	X	X	X
*Xenelmoides*									
**Total**	48	27	29		25	20	17	20	27

^+^Genus with larva already described.

*Associated larva in the process of description.

^X^Present in.

Other genera with unknown larvae found in neighboring countries (Table 1) correspond to the Amazonian fauna, and therefore are not present in western Colombia; consequently, they cannot be associated with the larvae described in this contribution.

## Material and methods

**Figure 1:**
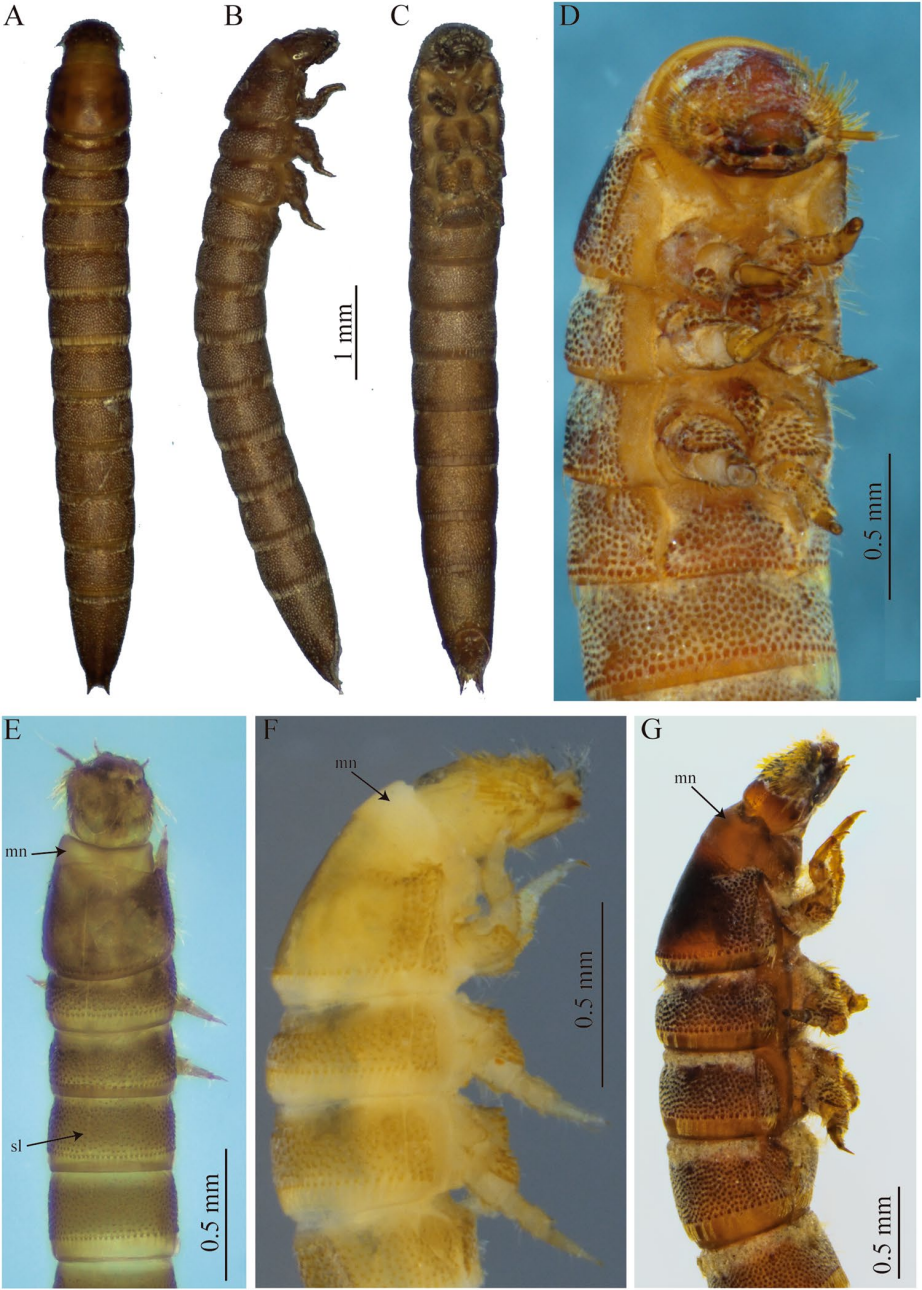
Habitus and color variation of *Stenhelmoides rufulus* larvae. (**A–C**) habitus, (**A)** dorsal, (**B**) lateral, (**C**) ventral views, (**D–G**) coloration (**D**) ventral, (**E**) dorsal, (**F**,**G**) lateral views. mn: membranous neck, sl: sagittal line.

Specimens were captured with a Surber net, killed and preserved in a 75% ethyl alcohol solution. Larval specimens were cleared using warm lactic acid and dissected and mounted on glass slides with PVLG medium. Observations were made using a Leica S6D dissecting microscope and a Leica DMLB compound microscope, both equipped with photographic cameras. Drawings of setae were scanned and digitally edited. Additional photographs (Fig. 1D,F,G) were taken with a digital camera Nikon DS-Ri1 U3, adapted to a stereoscope Nikon SMZ-1500. The photos were assembled using the freeware programs CombineZP^24^ and Helicon Focus®. The larval morphology nomenclature of Harris^25^, Shepard^26^, Borror *et al*.^27^, Lawrence^28^ and Kodada *et al*.^1^ were followed for the descriptions. Data on physical and chemical parameters of *Stenhelmoides* habitats were provided by the Water Laboratory of Institute for Research and Development in Water Supply, Environmental Sanitation and Water Resource Conservation - CINARA (by its acronym in Spanish) of Universidad del Valle (CINARA, unpublished data).

### Repositories

MUSENUV = Museo de Entomología de la Universidad del Valle, Cali, Colombia.

CLCH = Colección Limnológica de la Universidad Tecnológica del Chocó, Quibdó, Colombia.

CMA-UCO = Colección de Macroinvertebrados Acuáticos de la Universidad Católica de Oriente, Rionegro, Colombia.

COMAC-SINCHI = Colección de Macroinvertebrados Acuáticos de la Amazonía Colombiana, Leticia, Colombia.

IAvH-E = Colección Entomológica del Instituto de Investigación de Recursos Biológicos Alexander von Humboldt, Villa de Leyva, Colombia.

## Results

### Diagnosis of *Stenhelmoides* Grouvelle, 1908 mature larva

**Figure 2:**
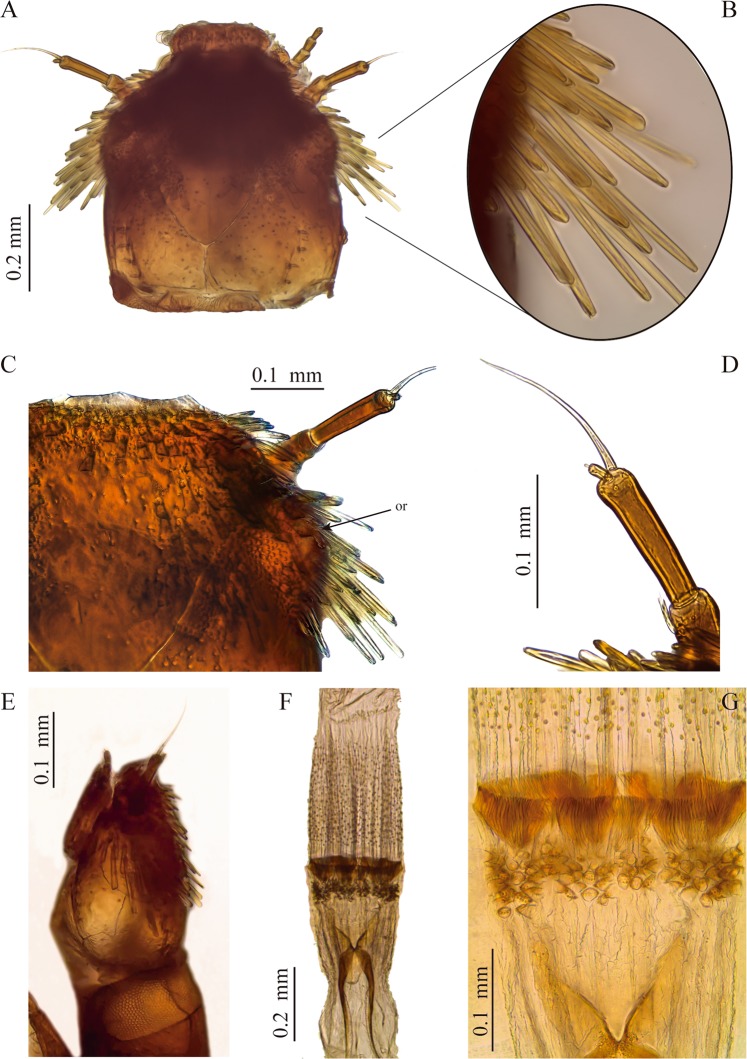
Head and mouthparts of *S. rufulus* larva. (**A–C**) Head, (**A**) dorsal view, (**B**) lateral spatulate setae, (**C**) frons and ocular region, (**D**) antenna, (**E**) head, lateral view, (**F,G**) proventriculus. or: ocular region.

**Figure 3:**
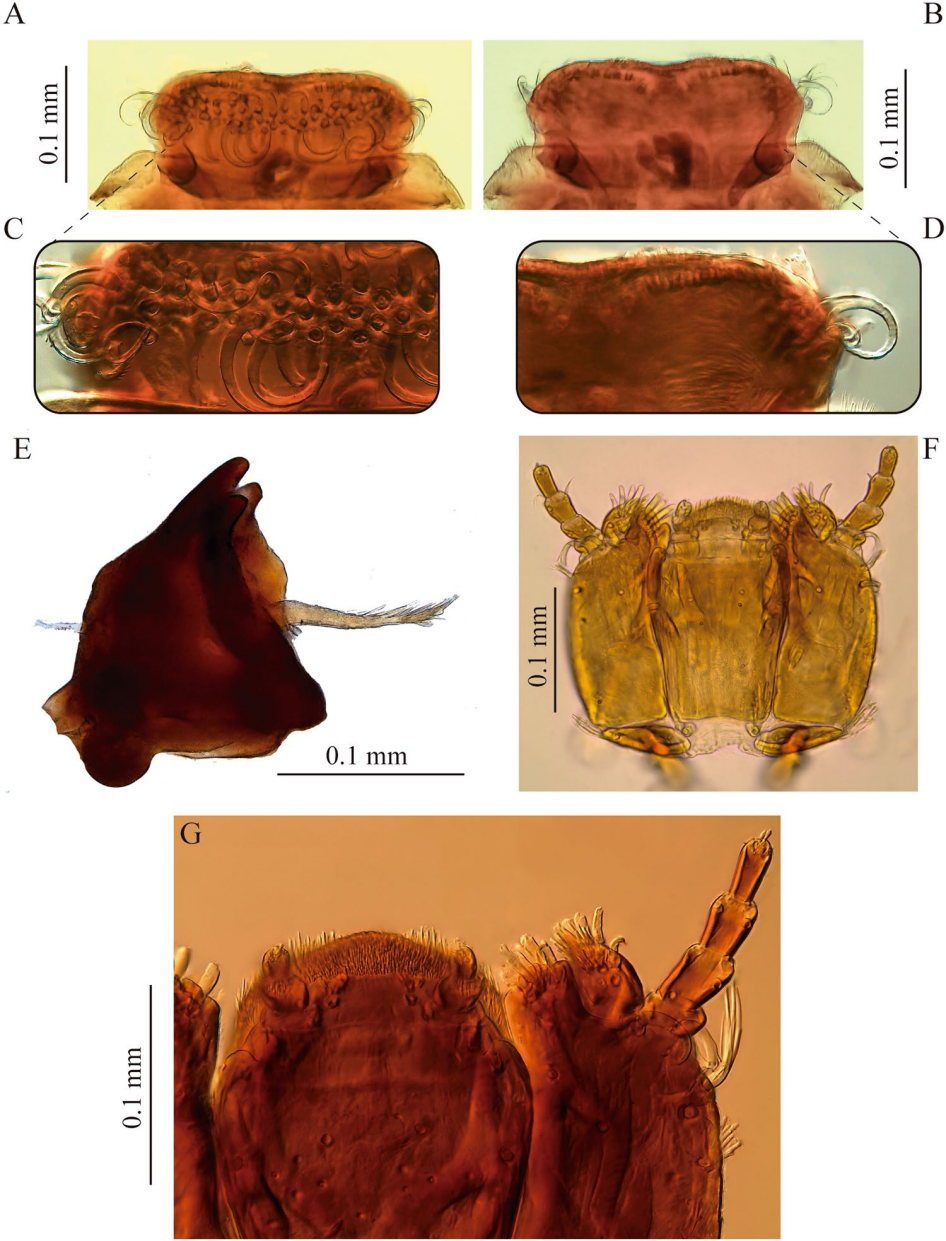
Mouthparts of *S. rufulus* larva. (**A-D**) labrum, (**A**) dorsal view, (**B**) ventral view, (**C**) dorsal detail, (**D**) ventral detail, (**E**) mandible, (**F-G**) maxillolabial complex.

**Figure 4:**
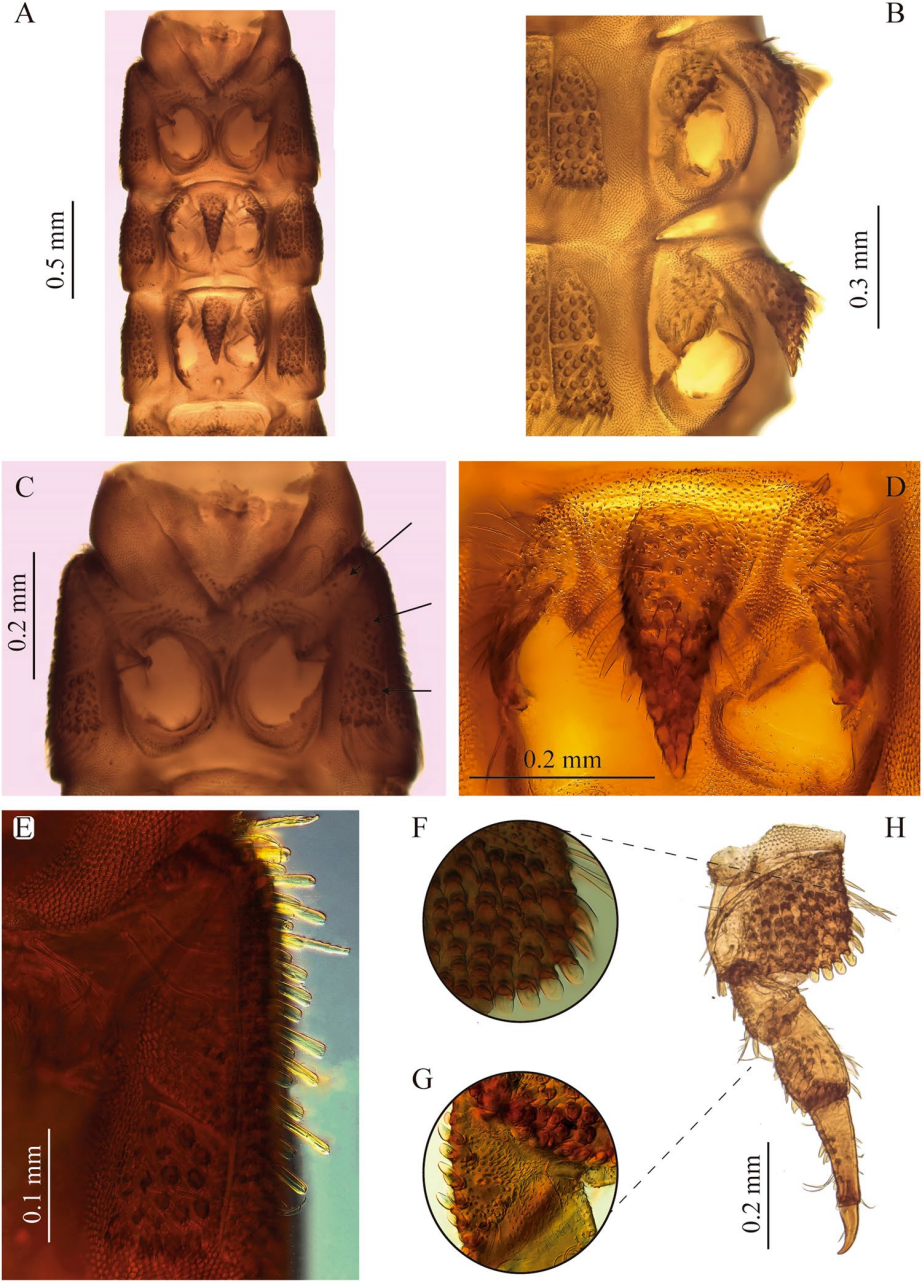
Thorax and legs of *S. rufulus* larva. (**A**) Thorax, ventral view, (**B**) meso- and metathorax, lateral view, (**C**) prosternum, (**D**) mesosternal process (**E**) prosternal sclerites, (**F**) coxa, (**G**) trochanter, (**H**) metathoracic leg. Arrows show prosternal sclerites.

**Figure 5:**
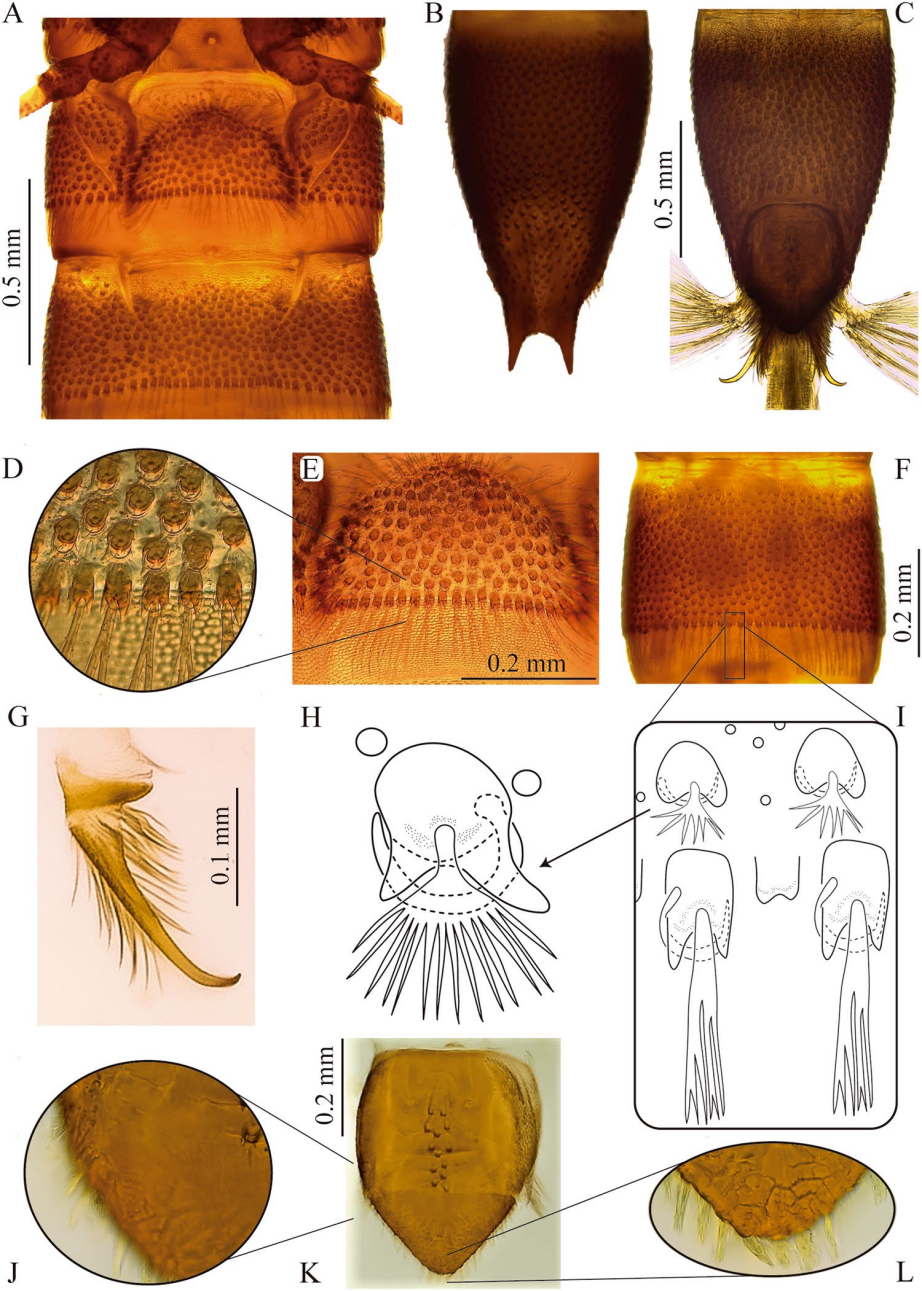
Abdomen of *S. rufulus* larva. (**A**) Abdominal sternites I – II, (**B,C**) abdominal segment IX, (**B**) dorsal, (**C**) ventral, (**D,E**) abdominal segment I, (**D**) sternum detail (**E**) posterior margin (**F**) abdominal segment IV, without sternal sutures in ventral view, (**G**) anal hook, (**H**) fan-shaped-tufted seta, (**I**) ramose setae, posterior margin of an abdominal segment, (**J–L**) anal operculum, (**J**) lateral margin, (**K**) external view, (**L**) apex.

Body elongate, subcylindrical, parallel sided; color yellowish brown to reddish brown; tegument surface with several spatulate and scale-like setae as long as antennae (excluding sensorium), especially on head, sternal region of thorax, legs and first abdominal sternite **(**Figs. 1–5**)**. Head exposed or partially concealed by pronotum, capable of being retracted into prothorax; frontal lines Y-shaped, merging basally into a long coronal line, about 1/4 of head; stemmata absent; clypeus concave without anterolateral tooth; labrum oval, wider than long; antenna with three antennomeres, second antennomere longer than other two together, with antennal sensorium long, as long as or slightly longer than second antennomere; mandibles symmetrical, with three apical teeth; prostheca long; maxilla with stipes longer than palpus, with four palpomeres; galea entire; lacinia well-developed, fused to stipes; labium with postmentum three times longer than prementum, palpi with two palpomeres, inserted on a short palpiger. Prothorax with six ventral sclerites, one antero-lateral pair and two lateral pairs, procoxal cavities open and membranous; meso- and metathorax with seven ventral sclerites, three large anterior process-like sclerites and two lateral smaller pairs, coxal cavities open; meso- and metathoracic sternal sclerite strong and prominent between coxae. Abdomen with a single pair of pleural sclerites on first segment; sternopleural and tergopleural sutures incomplete on second abdominal segment; abdominal segments wider than long; segment IX ending in two spinous processes with an emargination in the middle. Ventral operculum on abdominal segment IX pentagonal. Abdominal hooks lacking teeth on inner margin.

### Description of mature larva of *Stenhelmoides rufulus* (Hinton, 1934)

#### Body

Elongate, cylindrical, sides subparallel, thorax slightly wider than abdomen (Fig. 1). Color yellowish brown to reddish brown (Fig. 1). Tegument profusely tuberculate with several kinds of setae: tufted, either short or long, branched, spatulate, spine-like and scale-like (Figs. 2B, 5D,H–I). Anterior region of head, venter of thorax, legs and sternum of first abdominal segment remarkably covered by strong spatulate and scale-like setae. Length: 6.8–8.8 mm; maximum width: 0.87–1.05 mm.

#### Head

Capable of retraction into the thorax. Head capsule subquadrangular (Fig. 2A) (slightly wider than long). Dorsal surface at basal half smooth, except by some scattered punctures and two sublateral rows with four short setae lined on basal fourth (parietal area). Dorsal surface of anterior half coated with abundant, long, translucent, spatulate setae projected posteriad, more prominent on the sides. Coronal line long, about 1/4 as long as head capsule. Frontal lines long, extending to inner margin of antennal sockets. Frontoclypeal suture present. Frons protruding beyond antennae, with rounded anterolateral edges and anterior margin almost straight, anterior region without teeth. Ocular region on each side of head lighter in color and closer to base of antenna than to posterior end of head capsule. Lacking stemmata, but some individuals with one or two ocular pigment granules without lens.

#### Antenna

As long as half of head capsule (Fig. 2A,D). Basal antennomere (A1) short, slightly wider than long, bearing a crown of ramose setae. Second antennomere (A2) the longest, 1.5 times longer than first, bearing a distal long and prominent sensorium; slightly longer than second antennomere. Third antennomere (A3) the shortest, about 1/8 as long as second antennomere, bearing a tiny apical seta.

#### Mouthparts

Labrum subrectangular, convex, slightly wider at distal third (Fig. 3A,B). Anterior margin emarginated at middle, more evident from ventral view. Anterolateral angles rounded. Dorsal surface with a transverse band of strong long curled plumose setae arranged at about distal third. Ventral side with three stout simple setae on anterolateral corners, anterior margin with short simple setae with large sockets. Rest of ventral surface (epipharynx) covered by a dense pubescence oriented mesad and posteriad.

#### Mandibles

Strongly sclerotized, symmetrical, as long as wide, apex with three blunt teeth (Fig. 3E). Inner margin on dorsal side (dorsal carina) sharp, area between dorsal carina and mesal edge concave, with a brush of hair-like setae (pennicilus). Prostheca present, stout and plumose, shorter than mandible length, mesally oriented. Outer margin of dorsal surface with one tufted seta at about mid-length.

#### Maxilla

Basally with a transverse, narrow, suboval cardo, bearing a short tufted seta closer to outer margin (Fig. 3F,G). Stipes large, subrectangular, twice longer than wide; external (or ventral) surface of stipes with several setae distributed as follows: a longitudinal row of short tufted setae on outer margin, three short setae arranged as an oblique line from near base of lacinia to closer to outer margin and two long slender curled tufted setae near base of palpus. Lacinia and galea strongly sclerotized; lacinia subquadrangular, fully fused to stipes, apex truncated with several apical and subapical stout setae distally as well as on inner margin; galea entire, one-segmented, suboval, shorter than lacinia, bearing a group of stout distal setae. Palpus four-segmented, first palpomere the shortest with a curled seta; second palpomere twice as long as first, lacking setae; third palpomere slightly longer than second, with two apical setae, one on inner margin and one on outer margin; distal palpomere slightly shorter than second, bearing several short apical sensilla.

#### Labium

Well developed, formed by a large postmentum and a short prementum (Fig. 3F,G). Postmentum 1.5 times longer than wide, slightly narrower basally; anterolateral corners projected outwardly as lobes bearing a small digitiform sensillum; ventral surface with scattered pores clustered mainly on distal area of disc and two pairs of setae, one pair of short tufted setae at each distal corner and one pair on apical third, at each lateral margin; two small rounded sclerites near base of postmentum, bearing a long-stemmed tufted seta. Prementum short, wider than long, with a basal membranous region and an apical sclerotized plate bearing the palpi, disc with two spatulate setae and distal margin densely setose. Palpus two-segmented, palpiger present, well sclerotized; both palpomeres subequal in length; first palpomere bearing several short setae on outer apical corner; second palpomere narrower, bearing several apical sensilla.

#### Proventriculus

Equipped with strongly sclerotized teeth arranged as follows (Fig. 2F,G): one anterior row of clustered laminar teeth followed by a posterior band forming a well-defined belt of suboval teeth bearing about four tiny teeth; whole structure wider than long.

#### Thorax

Strongly sclerotized (Figs. 1D–G, 4); tergal plates with sagittal lines and densely covered with setiferous tubercles, except prothorax, which is smooth and shiny with tuberculate zones confined to lateral portions; posterior margin of all thoracic segments with a row of very long ramose setae, as long as adjacent membranous area. Ventral region of all thoracic segments with large reticulate membranous areas between sclerites. Prothorax the longest segment, subtrapezoidal, wider basally, slightly notched near apical margin, with a conspicuous anterior membranous neck, three times wider than long; dorsal surface sparsely and finely puncticulate, except for lateral areas which are densely covered with coarse setiferous tubercles; lateral margins with large spatulate setae and two basal tufted setae. Ventral region of prothorax with six sclerites, two antero-lateral subtriangular poorly sclerotized sclerites extending from lateral margin to near outer margin of coxal cavity, with long, slender tufted setae; two lateral pairs, the anteriormost subtriangular with conical and spatulate setae and membranous area continuing over inner margin of this sclerite, and the posteriormost subrectangular with coarse setiferous tubercles, conical and tufted setae. Posterior margin of ventral region of meso- and metathorax with a median circular impression. Meso- and metathorax wider than long, dorsally covered with setiferous tubercles and with an anterior ring of rugulose sculpture; ventrally with seven sclerites, one large anterior median conical process-like sclerite rising from a poorly sclerotized trapezoidal base, and two sub-median smaller subtriangular sclerites at each side of median sclerite, and two lateral pairs, one anterior subtriangular and one posterior subrectangular; coxal cavities open. Mesothorax bearing a pair of lateral spiracles.

#### Legs

Five-segmented, subequal in chaetotaxy and size (Fig. 4F–H). Coxa the largest segment, strong and subrectangular, external surface forming a protruding plate apically crenulated; plate with spine-like and scale-like setae on the disc and scale-like setae on edges; distal margin with a ring of scale-like setae; basal margin with scattered tufted setae, a patch of spine-like setae, few simple setae and basal spine-like sculpturing; apical edge emarginated in front of trochanter with scale-like setae; inner surface with a few simple and tufted setae on disc and apical scale-like and spine-like setae. Trochanter subtriangular, slightly shorter than femur, most of the surface covered by small spines and several scattered scale-like setae and a few ramose setae located mainly on the disc and edges; external surface with two spine-like setae on apical edge, near dorsal area. Femur subrectangular wider than tibia; anterior region almost glabrous with a row of long tufted setae at external edge; posterior margin coated with prominent setiferous tubercles and scale-like setae, internal surface with three spine-like setae and apical long tufted setae, external surface without setae or with some scattered pores. Tibia longer than femur, subrectangular to subtrapezoidal (base wider than the apex), anterior region with a row of short tufted setae and one medial long curly tufted setae, posterior region with two patches (subbasal and subapical) of tubercles with scale-like setae and two rows of scattered small and long tufted setae, external surface with two subbasal scale-like setae and posterior spine-like sculptures. Pretarsus subtriangular with a tiny distal seta slightly shorter than claw; claw blunt-pointed slightly curved (obtuse angle) at apical third.

#### Abdomen

Ninesegmented (Fig. 1A–C), strongly sclerotized. Surface covered by bicuspidated tubercles bearing a fan-shaped-tufted seta and a curly seta surrounding the tubercle, such as in thorax (Fig. 5D,H,I). Anterior edge of each abdominal segment with spine-like sculptures developing gradually from the membranous area to sclerotized area with larger tubercles. Posterior margins with a belt of quadrangular bicuspidated tubercles bearing a long ramose seta intercalated with a smaller tubercle accompanied by a shorter simple seta (Fig. 5D,I).

Segments I-VIII subequal in length, wider than long, segments VII-VIII almost as long as wide, segment IX the longest, 2 times longer than segment VIII with basal half parallel-sided and distal half tapering toward apex (Fig. 5B,C). Abdominal apex with a U-shaped emargination bearing thick tufted setae and two apical spinous processes 1/4 as long as segment IX (Fig. 5B). These projections are bare but surrounded by various setal types on base. Spiracles present on segments I-VIII. Segment I with complete sagittal line, segments II-IX lacking sagittal line. Pleural sclerite fusiform, only present on first abdominal segment, wider basally and narrowing towards the apex, inner margin (sternopleural suture) rounded and protruding above the sclerite, external margin (tergopleural suture) oblique on the same plane as the rest of the segment (Fig. 5A). Sternal plate semicircular, wider than long with strong scale-like and tufted setae on edges (Fig. 5E). Sternopleural and tergopleural sutures complete on first segment, incomplete on second segment reaching the anterior half (Fig. 5A). Segments III to IX without defined sutures, as complete rings with texture and tuberculation similar in ventral and dorsal face (Fig. 5F).

#### Operculum

Subpentagonal, longer than wide (Fig. 5K). Basal two thirds quadrate, lateral margins with short setae, external surface with two to three longitudinal rows of bicuspid tubercles on disc, rest of surface glabrous (Fig. 5K). Distal third acute, profusely hairy with tufted setae longer than those of lateral margins (Fig. 5J–L). Inner surface densely coated with simple setae, lateral vertex with thick tufted setae.

Anal hooks sharp on apical fifth obtusely curved close to 90° (Fig. 5G). Hooks non-serrated, lacking teeth on lateral margin. External and inner surfaces with long setae, slightly longer at inner base. Basal sclerite of hook twice as wide as hook base, bearing long setae. Gills composed by three tufts of simple gill filaments (more than 20 in each tuft).

### Larval material examined

**COLOMBIA** • 1 specimen; **Caquetá;** Florencia; Morelia; El Dedo creek; Hacha basin river; 1°38′58.7″N, 75°38′34.7″W; 309 m a.s.l.; 3 feb. 2009; S. Gaspar; M. Peláez-Rodríguez legs; rock; MUSENUV • 1 specimen; **Chocó**; Quibdó; Tutunendo river; N 5°44′32″N, 76°31′17.22″W; 96 m a.s.l.; 21 sep. 2004; S. Sánchez leg; rock; CLCH • 2 specimens; **Córdoba**; Tierralta; Tuis-Tuis creek; 8°1′26.29″N, 76°4′53.18″W; 225 m a.s.l.; 24 oct. 2014; J. Garcés leg; MUSENUV • 1 specimen; **La Guajira**; San Juan del Cesar; Caracolí; Ranchería river; 10°57′13.6″N, 73°3′15.6″W; 465 m a.s.l.; 18 oct. 2009; C. Tamaris; G. Rúa; C. Guzmán legs; leaf litter; MUSENUV • 2 specimens; **Magdalena**; Aracataca; Tucurinca river; 10°41′16.3″N, 74°1′29.3″W; 500 m a.s.l.; 8 may. 2010; C. Tamaris; T. Sierra; G. Rúa legs; gravel; MUSENUV • 3 specimens; the same information except Ciénaga; Toribío river; 11°2′59.21″N, 74°13′3.79″W; 30 m a.s.l.; 9 jun. 2007; CMA-UCO code1438 • 1 specimen; **Valle del Cauca**; Buenaventura; La Nevera creek; Dagua basin river; 3°48′26″ N, 76°46′50″W; 360 m a.s.l.; 19 feb. 1999; M. del C. Zúñiga; S. Mosquera; G. Guevara legs; rock; MUSENUV.

### Adult material of *Stenhelmoides rufulus* examined

**COLOMBIA •** 1♀; **Caquetá**; Florencia; before Florencia; Hacha river; 1°38′47.9″N, 75°36′37.1″W; 385 m a.s.l.; 30 sep. 2013; N. Oviedo leg; COMAC-Code 0589/Coleop 0135 • 1 specimen; Vereda Paraiso; Paraiso river; Hacha basin river; 71°38′58″N, 75°37′45″W; 682 m a.s.l.; 31 oct. 2013; S. Gaspar; M. Peláez-Rodríguez legs; rock; MUSENUV • 1♀; **Chocó**; Quibdó; Tutunendo river; 5°45′58.27″N, 76°33′33.23″W; 36 m a.s.l.; 18 oct. 2003. CMA-UCO Code1143 • 1 specimen; **Córdoba**; Tierralta; Tuis-Tuis creek (E1); 8°1′26.29″N, 76°4′53.18″W; 225 m a.s.l.; 24 oct. 2014; J. Garcés leg; MUSENUV • 2 specimens; the same information except (E2); 8°1′49.21″N, 76°5′11.30″W; 204 m a.s.l.; MUSENUV • 1♀; **La Guajira**; Cerrejón; 21 jun. 1981; Surber ned; IAvH-Code 140959 • 1♀, 1♀; **Magdalena**; Aracataca; Tucurinca; Tucurinca river; 10°41′16.3″N, 74°1′29.3″W; 500 m a.s.l.; 8 may. 2010; C. Tamaris; T. Sierra; G. Rúa legs; gravel; MUSENUV.

### Habitat

**Figure 6:**
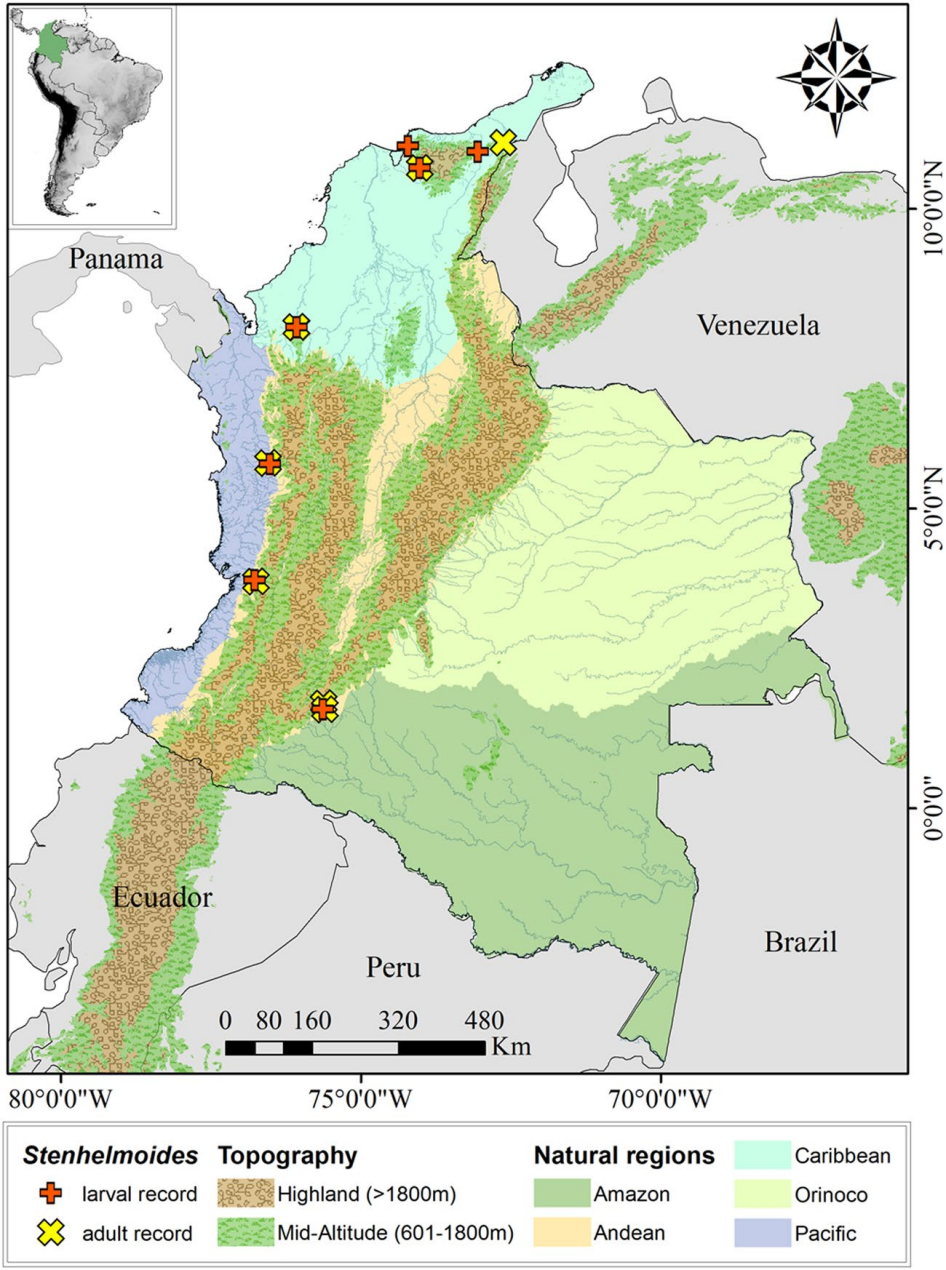
Map of the distribution of *Stenhelmoides* larval and adult records in this work.

**Figure 7:**
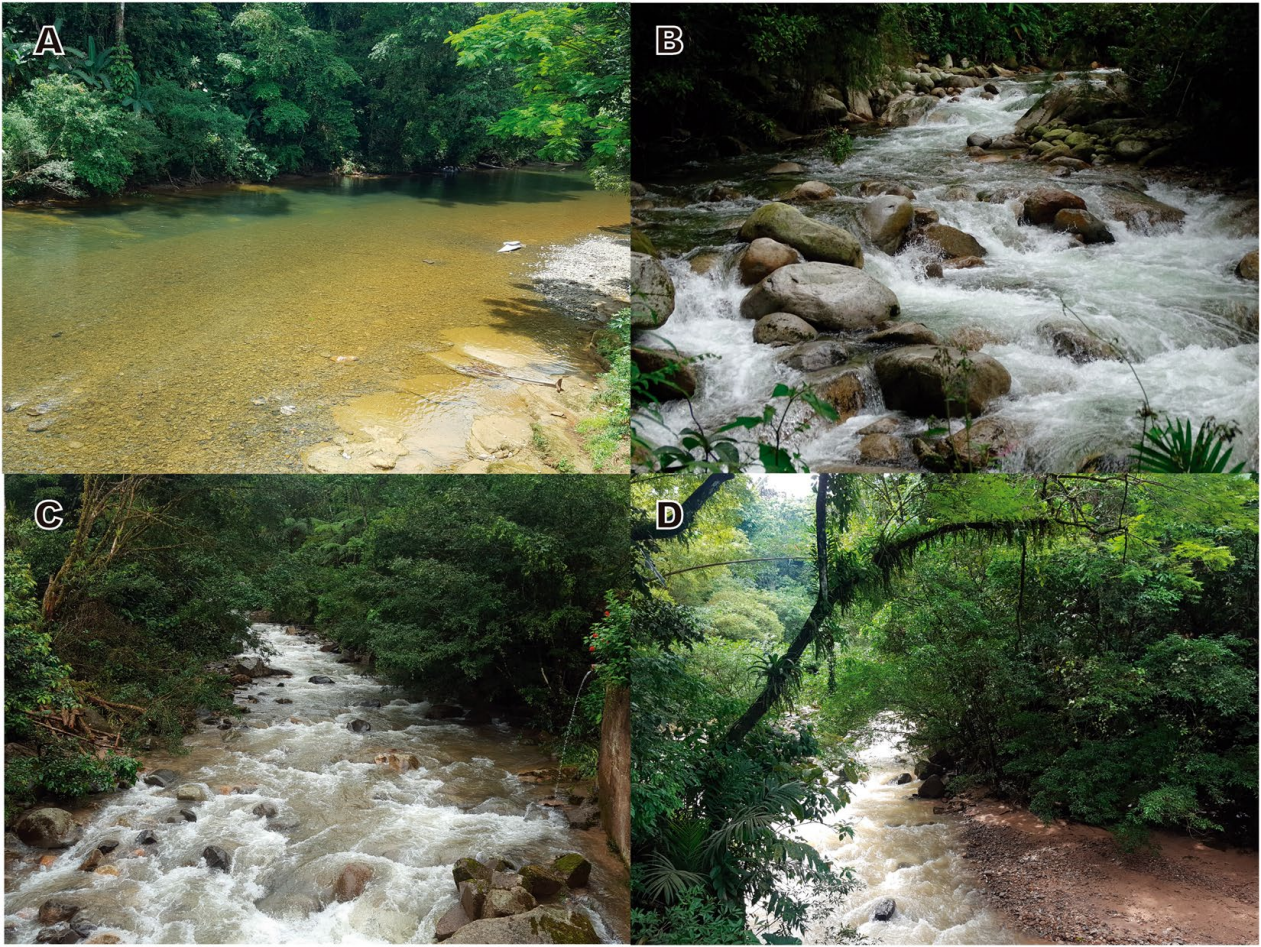
Sampling sites. (**A**) Tutunendo river, (**B**) Tucurinca river, (**C**) Paraiso River, (**D**) El Dedo creek. Photos: Zuleyma Mosquera (**A**); César Tamaris (**B**); Juliette Pauline Chaux (**C**,**D**).

Larvae and adults of *Stenhelmoides* were collected within rocky substrates and leafy litter situated in streams between 30 and 682 meters above sea level (m a.s.l.). The studied streams are located in three main natural geographic Colombian regions: the Pacific (two sites), the Caribbean (four sites), and the Andean portion along the foothill of the Amazon region (three sites) (Figs. 6 and 7A–D).

The Pacific region is located in the western portion of Colombia. The streams within this region belong to the macro-basins of the Atrato and Dagua rivers in the Choco Biogeographic ecoregion. This zone is recognized for its high biodiversity and profuse endemism. The predominant life zones are the tropical rain forest (bp-T) and the very humid tropical forest (bmh-T). In addition, this zone has an extensive hydric network due to intense rainfall, with some places reporting a multi-year average precipitation as high as 10,000 mm^29,30^.

The Caribbean region is located in the northern portion of Colombia and South America. The largest number of larvae and adults of *Stenhelmoides* were collected in this region, with sampling sites located in the foothills of different fronts of the Sierra Nevada de Santa Marta (SNSM) and in lowlands of the Valley of the Sinú River. The SNSM offers a rich hydric network with streams characterized by short lengths and significant altitudinal gradients due to the steep slopes and confined basins in the area. The predominant vegetation is associated with the humid subtropical forest (bh-BT), mostly present in forests along poorly preserved riverbanks. The deforestation in the zone has been favored to promote agricultural activities, which has generated great pressure on the hydrographic basins^31^.

The Andean-Amazonian transition is located in the southern portion of Colombia and the collection sites correspond to the Department of Caquetá. The foothills of this mountain range have the sub-Andean forest as the primary vegetation class, which gradually merges with the upper layer of the high-Andean forest and the lower layer of the humid forest of warm floors. These ecosystems have special ecological conditions associated with the transitional environment between the fauna of Andean origin and the fauna of the Amazon plains. Despite having their own characteristics, the vegetation layers gradually mix due to the changes in the altitudinal gradients. The sub-Andean forest has been fragmented over the last decades and has been affected by agricultural activities, which has resulted in the loss of large areas of forest and the deterioration of the quality of the water bodies^32^.

The sampling events were carried out in a heterogeneous group of water currents with different flow rates (Fig. 7). Three of them correspond to creeks with flows considered of low-order magnitude, generally between 0.25 and 0.80 m^3^.sec^−1^, and with abundant areas of rapids and rocky beds. Other six sites are located in rivers with medium- to high-order magnitude flow rates. Specifically, the Tutunendo river is the water body with the highest flow rate in the study, reporting a medium 157.67 m^3^.sec^−1^, a minimum 57.58 m^3^.sec^−1^, and a maximum 257.58 m^3^.sec^−1^ monthly multi-year average flows. In addition, the Hacha river reported a medium 34.5 m3/sec, a minimum 19.33 m^3^.sec^−1^, and a maximum 156.92 m^3^.sec^−1^ monthly multi-year average flows, while the Rancheria river reported a medium 6.17 m3/sec, a minimum 3.24 m^3^.sec^−1^, and a maximum 35.71 m^3^.sec^−1^ monthly multi-year average flows^33^. Out of this group, the Tucurinca and Toribío rivers in the Sierra Nevada de Santa Marta are the water bodies with the lowest flow rate in the study, with point measurements between 1.5 to 2.1 m^3^.sec^−1^ at the sampling locations in the middle basins. The Paraiso river has no information available for the flow. These creeks and rivers belong to multiple hydrographic regions in Colombia that drain to the Atlantic and Pacific Oceans and the Amazon River macro-basin.

In general, the sampled streams had relatively clear fresh waters (19 to 22 °C), with dissolved oxygen levels close to saturation and with low residual organic loads. The perimeter areas of the riparian corridors exhibited a different type and magnitude of vegetation coverage, with a secondary forest condition in a relatively good conservation state. For those streams with available information, the water quality index values fluctuated between 50 and 74 units (ICA-FSN)^34^ which correspond to a good environmental quality category. The oxygen saturation levels between 87% and 100%, along with biochemical oxygen demands between 0.5 and 1.5 mg.LO_2_^−1^, indicate that organic contamination in the streams is low. The pH values between 6.8 and 7.9 units indicate the presence of a stable acid-buffer system, while the specific conductance levels between 25 and 140 μ.cm^−1^, reveal low mineralization of the waters in the studied streams^31,35^ (CINARA, unpublished data). Although the distribution and substrates of larvae and adults were similar for the specimens collected as part of this investigation, both stages do not seem to share the same microhabitat.

### Associated Fauna

In this study, *Stenhelmoides* was found co-occurring with larvae and adults of *Austrolimnius formosus* (Sharp, 1882), *Cylloepus* Erichson, 1847, *Disersus* Sharp, 1882, *Heterelmis* Sharp, 1882, *Hexanchorus* Sharp, 1882, *Huleechius* Brown, 1981, *Macrelmis* Motschulsky, 1860, *Microcylloepus* Hinton, 1935, *Neocylloepus chaparensis* Manzo & Moya, 2010*, Neoelmis* Musgrave, 1935, *Phanocerus congener* Grouvelle, 1898, *Pharceonus volcanus* Spangler & Santiago-Fragoso, 1992 and *Xenelmis* Hinton, 1936.

## Discussion

### Comparative notes

Passos *et al*. diagnosed and illustrated “Elmidae larva D” in figures 19a and 19b as probably belonging to the genus *Stenhelmoides*^36^. That description matches with larvae described herein in the following: body elongated and cylindrical; integument covered by thick setae; head without frontal tooth; procoxal cavities open; pronotal disc without tubercles; pro- meso- and meta- thoracic pleural sclerites divided into two parts; pleural sclerites only on abdominal segment I; apex of abdominal segment IX with a pair of spinous processes and a emargination in the middle.

Since the larva of *S. rufulus* is the first one described for the genus, no other species of genus could be compared; therefore, it is compared with larvae of other genera that feature some of the same diagnostics characters.

Larvae of *Stenhelmoides* share the presence of a very long antennal sensorium on the second antennomere with the larvae of *Austrolimnius* Carter & Zeck, 1929. However, *Stenhelmoides* is different because it has a cylindrical body with uniform tuberculation, pleural sclerites defined only on first abdominal segment by compete sutures, sternopleural and tergopleural sutures incomplete on second abdominal segment, ninth abdominal segment with large spinous processes, presence of spatulate and scale-like setae on head and thorax and stemmata reduced.

*Stenhelmoides* larvae are also similar to those of *Cylloepus* and *Huleechius* in body shape and tegument; however, they are easily distinguished because they have a long antennal sensorium, frontal teeth absent, only one pair of pleurites on first abdominal segment and a pair spinous processes on the last abdominal segment with an emargination in the middle.

Larvae of *Typhloelmis* Barr, 2015 appear to be the most similar to those of *Stenhelmoides*. Both genera share a cylindrical body form, stemmata absent, antennal sensorium very long, large membranous areas between the thoracic sternal sclerites, coxal cavities open, thoracic pleuron divided in two pairs of sclerites, pleural sclerites present only on the first abdominal segment, and tip of the ninth abdominal segment emarginated with two dorsal lateral spinous processes. Conversely, the two genera can be easily distinguished because the second abdominal segment is ring-shaped since it lacks sternopleural sutures in *Typhloelmis*, while the sternopleural sutures extend to the second abdominal segment in *Stenhelmoides*. In addition, *Stenhelmoides* has strong translucent spatulate setae on the anterior region of head, thoracic sternum, legs and first abdominal ventrite, rather than the pale spines present in *Typhloelmis*. In *Stenhelmoides*, the setiferous tubercles of the tegument are denser than those of *Typhloelmis*, about 0.5–1 times their diameter instead of 1–2 times. The larvae of *Stenhelmoides* also have a median conical sternal process bearing stout scale-like spatulate setae, while the sternum seems to be membranous in *Typhloelmis*. Adults of *Typhloelmis* and *Stenhelmoides* share some similarities, such as a dorsal plastron, an oblong shape and lack of carinas in pronotum and elitra^14^.

Considering the similarities between adults and larvae of *Stenhelmoides* and *Typhloelmis*^14^ a similarity in the microhabitat of the larva is hypothesized. Consequently, larvae of *Stenhelmoides* with stemmata absent (or reduced to one ocular pigment lacking lens) and body coating of strong setae, could inhabit hyporheic zones or shelters formed between the rocks.

According to the larval key of South America^37^, *Stenhelmoides* does not key out because there is a conflict in couplet 4. The first option is *Cylloepus*, with cylindrical body and sternopleural suture reaching the abdominal segment IX. The second option includes several other body shapes, except cylindrical. *Stenhelmoides* does not match with any option; therefore, two new couplets are proposed in order to update the key:

4Body shape cylindrical.....................................................................................................................................4_a_

4′Body shape not cylindrical................................................................................................................................5

4_a_Sternopleural sutures reaching the ninth abdominal segment; anterior region of head with a pair of prominent teeth...................................................................................................................................*Cylloepus*

4_a_′Sternopleural sutures reaching the second abdominal segment; anterior region of head without teeth..............................................................................................................................................*Stenhelmoides*

Similarly, in the larval key of Neotropics^38^, *Stenhelmoides* does not key out, because there is a conflict in couplet 5. The first option includes larvae with a short antennal sensorium, while the second option is *Austrolimnius* with a long antennal sensorium and pleural sclerites on abdominal segments I to VII. *Stenhelmoides* does not match with any character, therefore to update the key two new couplets are proposed:

5(4)Second antennomere sensory appendage short, <second antennomere...................................................6

5′Second antennomere sensory appendage very long, ≥ second antennomere..........................................5_a_

5_a_(5′)Abdominal segments I-VII with pleural sclerites; tubercles on thoracic and abdominal segment arranged in a variable number of longitudinal rows...............................................................*Austrolimnius*

5_a_′Only first abdominal segment with pleural sclerites; tubercles on thoracic and abdominal segments not arranged in longitudinal rows....................................................................................................*Stenhelmoides*
